# Social Deprivation and Multimorbidity Among Community-Based Health Center Patients in the United States

**DOI:** 10.5888/pcd21.240060

**Published:** 2024-09-26

**Authors:** Steele Valenzuela, Katherine D. Peak, Nathalie Huguet, Miguel Marino, Teresa D. Schmidt, Robert Voss, Ana R. Quiñones, Corey Nagel

**Affiliations:** 1 Department of Family Medicine, Oregon Health & Science University, Portland; 2 OHSU-PSU School of Public Health, Oregon Health & Science University, Portland; 3 Research Department, OCHIN Inc., Portland, Oregon; 4 College of Nursing, University of Arkansas for Medical Sciences, Little Rock; 5 College of Public Health, Department of Biostatistics, University of Arkansas for Medical Sciences, Little Rock

## Abstract

**Introduction:**

Multimorbidity — having 2 or more chronic diseases — is a national public health concern that entails burdensome and costly care for patients, their families, and public health programs. Adults residing in socially deprived areas often have limited access to social and material resources. They also experience a greater multimorbidity burden.

**Methods:**

We conducted a retrospective cohort analysis of electronic health record (EHR) data from 678 community-based health centers (CHCs) in 27 states from the Accelerating Data Value Across a National Community Health Center (ADVANCE) Network, a clinical research network, from 2012–2019. We used mixed-effects Poisson regression to examine the relationship of area-level social deprivation (eg, educational attainment, household income, unemployment) to chronic disease accumulation among a sample of patients aged 45 years or older (N = 816,921) residing across 9,362 zip code tabulation areas and receiving care in safety-net health organizations.

**Results:**

We observed high rates of chronic disease among this national sample. Prevalence of multimorbidity varied considerably by geographic location, both within and between states. People in more socially deprived areas with Social Deprivation Index (SDI) scores in quartiles 2, 3, and 4 had greater initial chronic disease counts — 17.1%, 17.7%, and 18.0%, respectively — but a slower rate of accumulation compared with people in the least-deprived quartile. Our findings were consistent for models of the composite SDI and those evaluating disaggregated measures of area-level educational attainment, household income, and unemployment.

**Conclusion:**

Social factors play an important role in the development and progression of multimorbidity, which suggests that an assessment and understanding of area-level social deprivation is necessary for developing public health strategies to address multimorbidity.

**SUMMARY**

**What is already known on this topic?**

Evidence suggests that people living in communities with high levels of social and material deprivation are at greater risk for the development of multimorbidity (multiple chronic conditions), although few studies have been conducted among US populations.

**What is added by this report?**

Areas with greater levels of social and material deprivation were associated with higher rates of multimorbidity among a large cohort of patients served by community-based health centers in 27 US states. The multimorbidity burden, both within and across states, varied substantially.

**What are the implications for public health practice?**

Consideration of area-level social determinants of health is necessary to inform effective interventions to prevent or delay multimorbidity.

## Introduction

Multimorbidity, having 2 or more chronic diseases, is a growing public health issue that affects more than 50% of Americans aged 65 years or older and 80% of adults aged 85 years or older (1). Multimorbidity increases health care use, accounting for 78% of all clinical consultations in high-income countries (2). Poorly managed multimorbidity can have substantial consequences for health-related quality of life, threaten functional independence, and contribute to premature death (1,3). Multimorbidity is unequally distributed by age, race, ethnicity, sex, education, income, and geographic residence and is therefore, particularly important to evaluate within a socio-ecological model of health (4,5). The importance of place is further emphasized by the Healthy People 2030 goals, in which 2 of the 5 key social determinants of health address area-level factors, such as social and community context, neighborhood composition, and the built environment (6–8). Identifying environmental contributors to chronic disease development can help mitigate the multimorbidity burden.

A robust body of literature documents the contribution of area-level factors in the development and progression of individual chronic diseases. Overwhelming evidence shows that residing in areas with low levels of material and social resources increases the risk of developing and exacerbating chronic diseases (6,8). In particular, residents of areas with high social deprivation (eg, educational attainment, household income, unemployment) are at heightened risk for obesity, cardiovascular disease, and respiratory conditions (6). 

People living in areas with the highest levels of social deprivation have 3.7 times higher odds of having multimorbidity with a 10-year earlier onset than those from less deprived areas (2,9). People living in highly socially deprived neighborhoods, as they age, may be limited to resources available in their immediate environment and, as a consequence, be more vulnerable than younger adults to environmental stressors. This heightened vulnerability may be exacerbated by health-related consequences of environmental stress, such as depression and disability (10).

Community-based health centers (CHCs) are an essential component of the country’s network of safety-net clinics and are a vital resource for people living in under-resourced communities. CHCs provide health care to 10 million middle-aged and older patients annually and primarily serve socially deprived communities, people in living in poverty, and people from racial and ethnic minority communities (11). CHC patients have more complex medical diagnoses and develop advanced multimorbidity at comparatively younger ages than non-CHC patients (11,12). Equipping these clinical sites — which serve a large proportion of patients from socially deprived areas — with a full understanding of the role the neighborhood plays in the development and progression of disease would better equip them to provide health care to an increasingly complex patient community.

 Although previous studies found higher levels of social deprivation to be associated with greater disease burden and earlier onset of chronic disease (2,9), few studies mapped the distribution of multimorbidity and area-level social deprivation across geographic regions. Fewer still focused on CHC patients.

Our study described the burden of multimorbidity and evaluated chronic disease accumulation (ie, increase in a person’s count of chronic diseases over time) among middle-aged and older CHC patients, stratified by an area-level Social Deprivation Index (SDI) across a wide geographic area of the US. We used electronic health record (EHR) data geographically linked with American Community Survey (ACS) (13) data to examine the multimorbidity burden, social deprivation, and individual community-level factors. To our knowledge, ours is the first study to link EHR and ACS data to assess the geographic distribution of multimorbidity in a national clinical research network (CRN) for people in the US who rely on the health care safety net.

## Methods

### Data source

EHR data were obtained from the Accelerating Data Value Across a National Community Health Center (ADVANCE) Network, a multistate CRN in the National Patient-Centered Clinical Research Network (14). ADVANCE is led by OCHIN (https://ochin.org/solutions/operations/) in partnership with Health Choice Network, Fenway Health, and Oregon Health & Science University (OHSU). ADVANCE contains the largest collection of EHR data from patients receiving care in US safety-net facilities. The patient population is representative of the US CHC population (11,15). The study’s protocol was approved by OHSU’s Institutional Review Board (ID no. STUDY00020124).

### Study population

We assessed data from a retrospective rolling cohort of patients receiving care in 678 CHCs across 27 states from 2012, the first year of complete patient data in ADVANCE, through 2019, the starting date of our current study. A total of 816,921 patients met the study inclusion criteria: aged 45 years or older by study’s end and had a total of 2 or more clinician office visits in 2 or more separate years (16).

### Zip code tabulation areas (ZCTAs)

Our geographic unit of analysis, the zip code tabulation area (ZCTA), was assigned to patients based on the zip code area where they had resided longest, based on the date of the first reported ZCTA to the end date or date of a newly reported ZCTA. For patients with missing ZCTA end dates (last known date a patient resided in a ZCTA), we assigned their last clinical visit date as the end date. Start dates recorded before our study started in 2012 were truncated to January 1, 2012. For patients with a single start and end date, the mid date (year, month, and day) and corresponding mid year were determined. For patients with multiple start and end dates, their mid dates and mid years were determined. For example, if a patient recorded a single ZCTA from January 1, 2013, to January 2, 2017, their mid date was January 1, 2015, and 2015 was their ensuing mid-year. Lastly, we used rural–urban commuting area codes mapped to ZCTAs to classify each participant’s residential location as urban or rural (17).

### Dependent variable

Multimorbidity (defined as ≥2 chronic conditions) was assessed from 22 chronic diseases listed in the US Department of Health and Human Services Multiple Chronic Conditions framework (18) to identify multimorbidity: anxiety, arthritis, asthma, autism, cancer, cardiac arrhythmia, chronic kidney disease, chronic obstructive pulmonary disease, congestive heart failure, coronary artery disease, dementia, depression, diabetes, hepatitis, human immunodeficiency virus, hyperlipidemia, hypertension, osteoporosis, posttraumatic stress disorder, schizophrenia, substance use disorder, and stroke. We identified chronic diseases in patient charts by extracting diagnoses listed on patient encounter records and problem lists in the EHR. Prior work displayed good concordance across data sources for ascertaining diagnoses in this CHC network (19). For diagnosis codes located in encounter records, we followed the Chronic Condition Warehouse algorithms from the Centers for Medicare & Medicaid Services to ascertain disease status and date of onset (20). The number of chronic conditions was treated as a count variable (0–22) for summaries and modeling rates, and as a categorical variable (none, 0–1; low, 2–4; high, ≥5) for summaries and maps.

### Independent variables

**Social deprivation index (SDI) score.** SDI scores range from 1 to 100 and are a composite of 7 area-level variables collected by the US Census Bureau (21), including percentage of residents who live in poverty, rented housing units, overcrowded housing units, single-parent households, households without a car, people with less than 12 years of education, and unemployed adults aged younger than 65 years (22). We obtained 2 SDI estimates, in 2015 and 2017, that contained data over the 5-year spans: 2011–2015 and 2013–2017. Five-year intervals were used to increase the statistical reliability of the data for less populated areas and small population subgroups (13). We then averaged the SDI scores for the ZCTA where each patient resided the longest and generated quartiles based on the national distribution, with higher quartiles indicating greater social deprivation.

**Community-level characteristics.** In addition to SDI score, we examined discrete ZCTA-level indicators of median household income, percentage unemployment, and percentage college graduates, obtained from ACS. These indicators were calculated as 5-year moving averages from 2010 through 2018, where the value assigned to a given year was calculated as the average of that year, 2 preceding years, and 2 following years. For each indicator, we created a categorical variable using Jenks Natural Breaks (23). For indicators with skewed distributions or outliers, breaks were created by using the bottom 90% of ZCTAs, with the remaining top 10% of ZCTAs classified as the highest category. These were then linked to participant records based on the corresponding mid year of their longest resided ZCTA. If the mid year of the longest resided ZCTA occurred after 2016 (2017–2019), we extended 2016 community-level characteristics to these patients.

**Patient characteristics.** Patient characteristics were extracted from EHR data and included age at last visit (45–54 y, 55–64 y, ≥65y), sex, and a mutually exclusive categorical variable combining race or ethnicity and language (non-Hispanic Asian, non-Hispanic Black, non-Hispanic White, Hispanic preferring English, Hispanic preferring Spanish, Hispanic preferring another language, and non-Hispanic Other) to capture potential differences related to acculturation among Hispanic adults (24). Visit-based characteristics included insurance coverage patterns (continuously uninsured, continuously insured, discontinuously or intermittently insured) and federal poverty level (FPL) status (continuously <138%, continuously ≥138%, intermittently over or under 138%) across the study period.

### Statistical analysis

We summarized patient- and community-level indicators with counts and percentages for categorical variables and means and SDs for continuous variables. Indicators were then stratified by SDI quartile groups. We conducted *t* tests and χ^2^ tests to assess statistical differences between SDI quartile groups, with a value of *P* < .01 indicating significance.

We generated ZCTA-level graphical summaries of national and state maps to display geographic variability in the number of chronic conditions (averaged within each ZCTA). To assess the association between SDI quartile and chronic disease accumulation, we estimated longitudinal mixed-effects Poisson regression models. We included random effects to account for repeated measures at the patient level and the clustering of patients within states. Two separate models consisted of 1) an aggregate SDI model (a model that included patient-level covariates, time in years, SDI quartile groups, and an interaction effect between time and SDI quartile to examine differences in the rate of chronic disease accumulation between SDI quartile groups); and 2) a disaggregated model that included discrete community-level indicators of social deprivation but included no interaction effects with time. We exponentiated the coefficients of model-fixed effects to report incidence rate ratios (IRRs) and calculated corresponding 99% CIs. We tested for collinearity by estimating the variance inflation factor of all patient-level covariates.

For additional context, we also constructed 1) bivariate models of the association of baseline chronic condition count and community-level indicators; 2) the number of instances patients moved between ZCTAs and which SDI quartiles they moved between; 3) state-level characteristics, including number of clinics, number of patients, patients per ZCTA, number of ZCTAs, and mean number of chronic diseases; and 4) SDI-level characteristics by state, including the total number of ZCTAs and the mean number of chronic diseases displayed as multimorbidity categories (Appendix). All analyses were conducted using R version 4.3.1 (R Foundation), with the lme4 package used for model estimation and the ggplot2, ggnewscale, sf, and tigris packages used to generate maps.

## Results

The overall sample was 57.5% female, 38.8% non-Hispanic White, 17.6% non-Hispanic Black, 23.4% Hispanic preferring Spanish, and 51.0% continuously insured (Table 1). By the end of the study period, 37.4% of patients were aged 55 to 64 years. The mean number of chronic diseases recorded was 1.04 (SD = 0.9) at the beginning and 1.86 (SD = 1.6) at the end of the study.

**Table 1:** Patient (N = 816,921) Characteristics, Study of Social Deprivation and Multimorbidity Among Community-Based Health Center Patients, by SDI Quartile, ADVANCE Clinical Research Network, 2012–2019

Characteristic^a^	Total	SDI quartile 1	SDI quartile 2	SDI quartile 3	SDI quartile 4
**Number of patients**	816,921 (100.0)	53,121 (6.5)	145,233 (17.8)	226,945 (27.8)	391,622 (47.9)
**Age at last visit, y**					
45–54	293,762 (36.0)	17,063 (32.1)	49,111 (33.8)	79,450 (35.0)	148,138 (37.8)
55–64	305,394 (37.4)	19,207 (36.2)	53,453 (36.8)	84,414 (37.2)	148,320 (37.9)
≥65	217,765 (26.7)	16,851 (31.7)	42,669 (29.4)	63,081 (27.8)	95,164 (24.3)
**Sex**					
Female	469,733 (57.5)	30,521 (57.5)	83,569 (57.5)	130,542 (57.5)	225,101 (57.5)
Male	347,188 (42.5)	22,600 (42.5)	61,664 (42.5)	96,403 (42.5)	166,521 (42.5)
**Race or ethnicity**					
Non-Hispanic Asian	28,048 (3.4)	1,749 (3.3)	4,379 (3.0)	6,616 (2.9)	15,304 (3.9)
Non-Hispanic Black	143,677 (17.6)	3,536 (6.7)	10,565 (7.3)	25,650 (11.3)	103,926 (26.5)
Hispanic, preferred language					
English	77,381 (9.5)	3,661 (6.9)	10,528 (7.2)	21,621 (9.5)	41,571 (10.6)
Spanish	191,002 (23.4)	4,472 (8.4)	20,600 (14.2)	43,779 (19.3)	122,151 (31.2)
Another language	1,574 (0.2)	77 (0.1)	242 (0.2)	390 (0.2)	865 (0.2)
Non-Hispanic White	317,159 (38.8)	34,230 (64.4)	85,982 (59.2)	111,539 (49.1)	85,408 (21.8)
Non-Hispanic Other	9,271 (1.1)	550 (1.0)	2,228 (1.5)	3,055 (1.3)	3,438 (0.9)
Unknown	48,809 (6.0)	4,846 (9.1)	10,709 (7.4)	14,295 (6.3)	18,959 (4.8)
**Insurance coverage**					
Continuously uninsured	106,730 (13.1)	6,041 (11.4)	21,023 (14.5)	32,588 (14.4)	47,078 (12.0)
Continuously insured	416,236 (51.0)	29,747 (56.0)	74,440 (51.3)	113,199 (49.9)	198,850 (50.8)
Discontinuously insured	283,624 (34.7)	16,141 (30.4)	47,555 (32.7)	78,032 (34.4)	141,896 (36.2)
Missing	10,331 (1.3)	1,192 (2.2)	2,215 (1.5)	3,126 (1.4)	3,798 (1.0)
**Federal poverty level, %**					
Continuously <138	483,120 (59.1)	23,008 (43.3)	72,897 (50.2)	123,634 (54.5)	263,581 (67.3)
Continuously ≥138	85,292 (10.4)	8,586 (16.2)	19,800 (13.6)	28,706 (12.6)	28,200 (7.2)
Intermittently over or under 138	100,718 (12.3)	6,047 (11.4)	18,621 (12.8)	34,268 (15.1)	41,782 (10.7)
Missing	147,791 (18.1)	15,480 (29.1)	33,915 (23.4)	40,337 (17.8)	58,059 (14.8)
**Urbanity/rurality**					
Urban	646,209 (79.1)	39,340 (74.1)	107,835 (74.2)	157,966 (69.6)	341,068 (87.1)
Rural	170,712 (20.9)	13,781 (25.9)	37,398 (25.8)	68,979 (30.4)	50,554 (12.9)
**Longest resided ZCTA, y, mean (SD)**	5.5 (4.3)	4.7 (3.6)	5.4 (4.2)	5.6 (4.1)	5.6 (4.5)
**SDI Score, mean (SD)**	68.3 (24.4)	16.4 (6.3)	39.2 (7.1)	62.9 (6.9)	89.3 (7.4)
**SDI Score, median (IQR)**	73.0 (50.5–89.5)	18 (11.5–22.0)	40.5 (33.5–44.5)	64.0 (57.5–68.5)	90.5 (83.0–96.0)
**Visits per year, mean (SD)**	4.4 (5.5)	4.0 (4.4)	4.4 (6.5)	4.3 (5.7)	4.5 (5.1)
**Number of chronic conditions, mean (SD)**					
At patient’s first recorded visit	1.04 (0.88)	0.92 (0.81)	1.06 (0.94)	1.06 (0.93)	1.03 (0.84)
At patient’s last recorded visit	1.86 (1.61)	1.66 (1.54)	1.85 (1.63)	1.89 (1.66)	1.88 (1.57)
**Area-level indicators**					
No. of distinct ZCTAs	10,310	2,395	3,016	2,690	2,209
**Median annual household income, $**					
0 – <56,447	5,708 (55.4)	369 (15.4)	1,399 (46.4)	1,937 (72.0)	2,003 (90.7)
56,447 – <93,994	3,671 (35.6)	1,308 (54.6)	1,442 (47.8)	716 (26.6)	205 (9.3)
≥93,994	912 (8.8)	706 (29.5)	172 (5.7)	33 (1.2)	1 (0.0)
Missing	<11 (0.2)	<11 (0.5)	<11 (0.1)	<11 (0.1)	<11 (0)
**Unemployment, %**					
0 – <8.74	6,542 (63.5)	1,968 (82.2)	2,133 (70.7)	1,596 (59.3)	845 (38.3)
8.74 – <19.39	3,262 (31.6)	292 (12.2)	764 (25.3)	999 (37.1)	1,207 (54.6)
≥19.39	367 (3.6)	41 (1.7)	83 (2.8)	89 (3.3)	154 (7.0)
Missing	<11 (1.3)	<11 (3.9)	<11 (1.2)	<11 (0.2)	<11 (0.1)
**College graduates%**					
0 – <25.89	5,462 (53.0)	485 (20.3)	1,505 (49.9)	1,709 (63.5)	1,763 (79.8)
25.89 – <48.07	3,389 (32.9)	1,120 (46.8)	1,139 (37.8)	741 (27.5)	389 (17.6)
≥48.07	1,446 (14.0)	783 (32.7)	366 (12.1)	240 (8.9)	57 (2.6)
Missing	<11 (0.1)	<11 (0.3)	<11 (0.2)	<11 (0.0)	<11 (0)

Abbreviations: ADVANCE, Accelerating Data Value Across a National Community Health Center Network; IQR, interquartile range; SDI, Social Deprivation Index; ZCTA, zip code tabulation area.

a Values are n (%) unless otherwise indicated.

More than 75% of the sample resided in ZCTA’s in the highest 2 SDI quartiles, and 79.1% lived in primarily urban ZCTAs (Table 1). More than half of the sample (55.4%) resided in ZCTAs with a median household income of $0 to $56,447, a similar proportion (53.0%) lived in ZCTAs with the smallest number of college graduates, and 35.2% resided in ZCTAs with unemployment rates higher than 8.74%. Residential stability at the state (98.7%) and ZCTA-level (80.8%) was high, and among patients who remained in 1 state and moved between ZCTAs, nearly half (42.6%) stayed in the same SDI quartile (Table 2). Separately, 29.4% stayed in the most deprived quartile (quartile 4) and 21.3% moved between the 2 most deprived quartiles (quartiles 3 and 4).

**Table 2:** Patient Relocation and Movement Between States, ZCTAs, and Quartiles, ADVANCE Clinical Research Network, 2012–2019

Characteristic	N (%)
**Number of states resided**	
1	816,921 (98.7)
≥2	10,873 (1.3)
Total	827,794 (100.0)
**Number of ZCTAs recorded^a^**	
1	659,660 (80.8)
2	119,843 (14.7)
≥3	37,418 (4.6)
Total	816,921 (100.0)
**SDI quartile movement^a^**	
Remained in quartile 4	46,207 (29.4)
Moved between quartiles 3 and 4	33,559 (21.3)
Moved between quartiles 2 and 3	18,134 (11.5)
Moved between quartiles 2 and 4	15,389 (9.8)
Remained in quartile 3	12,237 (7.8)
Remained in quartile 2	6,528 (4.2)
Moved between quartiles 1 and 3	5,779 (3.7)
Moved between quartiles 1 and 2	4,777 (3.0)
Moved between quartiles 1 and 4	4,640 (3.0)
Moved between quartiles 2, 3, and 4	4,563 (2.9)
Remained in quartile 1	1,872 (1.2)
Moved between quartiles 1, 2, and 3	1,270 (0.8)
Moved between quartiles 1, 3, and 4	1,222 (0.8)
Moved between quartiles 1, 2, and 4	729 (0.5)
Moved between quartiles 1, 2, 3, and 4	355 (0.2)
Total	816,921 (100.0)

Abbreviations: ADVANCE, Accelerating Data Value Across a National Community Health Center; SDI, Social Deprivation Index; ZCTA, zip code tabulation area.

a ZCTA relocation and quartile movement are only reported for our patient sample whose records indicate they lived in 1 state.

When comparing across SDI quartiles, quartiles 1 (64.4%) and 2 (59.2%) had higher proportions of non-Hispanic White patients compared with quartiles 3 (49.1%) and 4 (21.8%) (Table 1). Quartiles 3 (54.5%) and 4 (67.3%) also consisted of higher proportions of patients continuously with incomes less than 138% of the FPL compared with quartiles 1 (43.3%) and 2 (50.2%). Patients residing in quartile 1 averaged 1.66 (SD = 1.54) illnesses at the end of the study, and the average for quartiles 2 to 4 ranged from 1.85 to 1.89 illnesses. Quartiles 1, 2, and 3 contained proportionally more patients residing in rural ZCTAs (25.8%–30.4%) than quartile 4 (12.9%), which comprised predominantly metropolitan/urban ZCTAs (87.1%).

**Figure 1:**
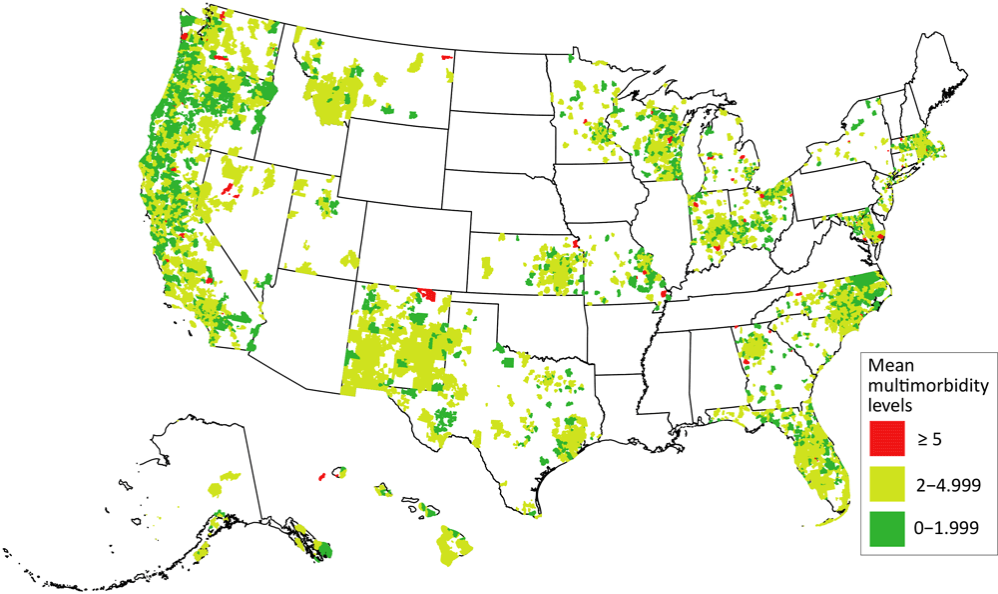
Mean multimorbidity levels for US, by ZCTA. Source: ADVANCE Clinical Research Network (14), 2012–2019. Abbreviation. ZCTA, zip code tabulation area.

**Figure 2:**
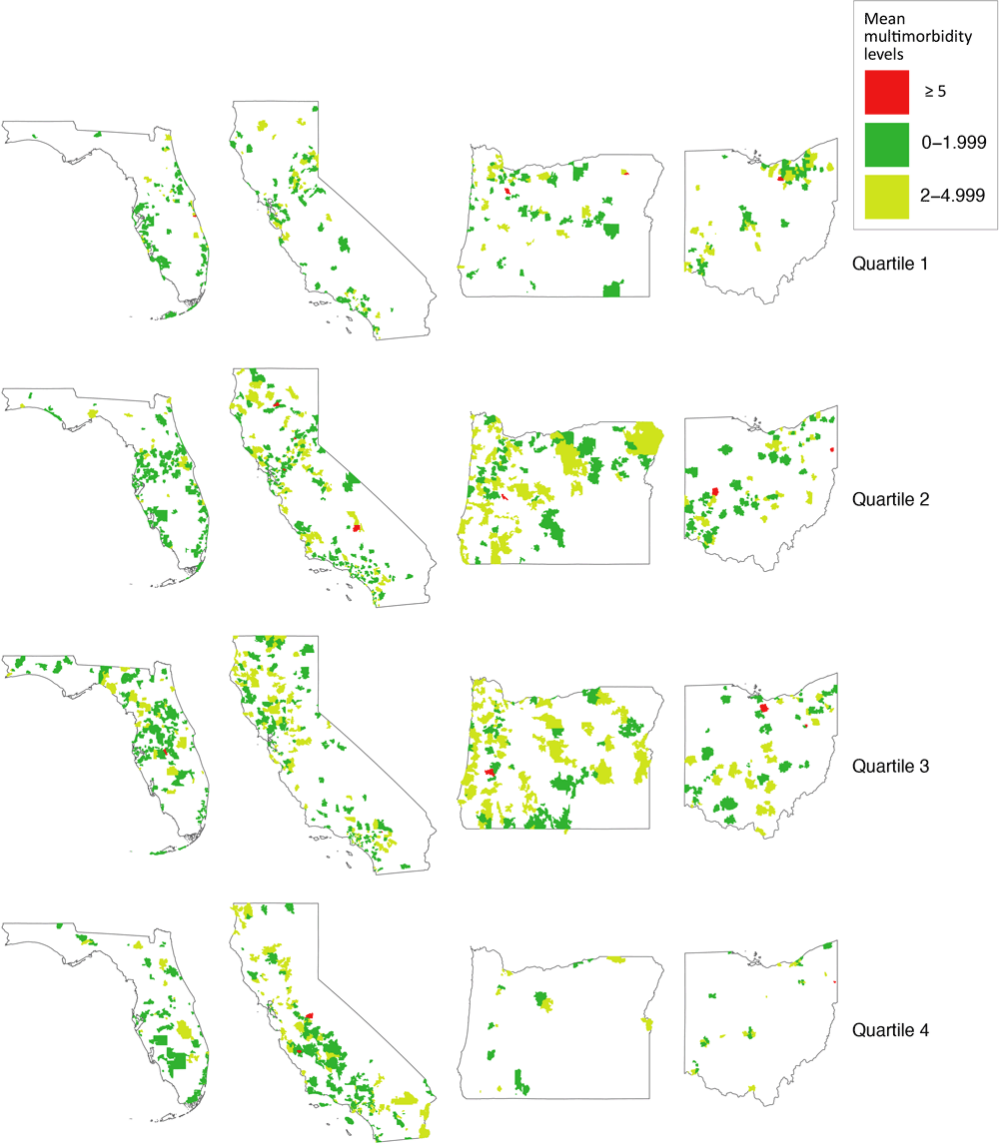
Distribution of mean multimorbidity levels by ZCTA for selected states (Florida, California, Oregon, and Ohio) and Social Deprivation Index quartiles. Source: ADVANCE Clinical Research Network (14), 2012–2019. Abbreviations: SDI, Social Deprivation Index; ZCTA, zip code tabulation area.

The mean number of chronic diseases ranged from 0.9 in Texas (SD, 1.1) to 2.2 in North Carolina (SD, 1.6) and Oregon (SD, 1.9) (Figure 1). Although our study consisted of 653 CHCs across a wide geographic area, 3 states (Florida, California, and Oregon) contributed 62.3% of our patient sample. Although multimorbidity prevalence varied across both states and ZCTAs, we saw no underlying pattern in the observed geographic distribution (Figure 2).

**Table:** 

State	Characteristics				
	No. of chronic diseases, mean (SD)	Clinics, N (%)	Patients, N (%)	ZCTAs, N (%)	Patients per ZCTA, mean (SD)
Alaska	1.6 (1.4)	1 (0.2)	1969 (0.2)	29 (0.5)	67.9 (355.4)
California	2.1 (1.6)	134 (20.5)	159,416 (19.5)	1,282 (20.0)	124.3 (404.9)
Connecticut	1.2 (1.4)	1 (0.2)	23 (0.0)	22 (0.3)	1.0 (0.2)
Florida	1.5 (1.3)	120 (18.4)	208,057 (25.5)	726 (11.3)	286.6 (555.6)
Georgia	1.3 (1.4)	11 (1.7)	9,805 (1.2)	195 (3.0)	50.3 (120.1)
Hawaii	1.7 (1.5)	5 (0.8)	7,403 (0.9)	46 (0.7)	160.9 (653.3)
Indiana	1.8 (1.4)	23 (3.5)	12,038 (1.5)	235 (3.7)	51.2 (122.5)
Kansas	1.4 (1.4)	3 (0.5)	3,864 (0.5)	131 (2.0)	29.5 (189.2)
Massachusetts	1.9 (1.6)	40 (6.1)	75,977 (9.3)	392 (6.1)	193.8 (676.6)
Maryland	1.8 (1.5)	15 (2.3)	13,151 (1.6)	164 (2.6)	80.2 (265.3)
Michigan	1.7 (1.1)	1 (0.2)	254 (0.0)	128 (2.0)	2.0 (1.8)
Minnesota	1.7 (1.6)	7 (1.1)	6,043 (0.7)	172 (2.7)	35.1 (108.7)
Missouri	2.0 (1.6)	4 (0.6)	8,185 (1.0)	153 (2.4)	53.5 (157.6)
Montana	1.8 (1.7)	6 (0.9)	7,420 (0.9)	84 (1.3)	88.3 (607.4)
North Carolina	2.2 (1.6)	22 (3.4)	26,693 (3.3)	342 (5.3)	78.0 (249.6)
New Jersey	1.2 (1.3)	1 (0.2)	47 (0.0)	38 (0.6)	1.2 (0.7)
New Mexico	1.7 (1.5)	37 (5.7)	43,919 (5.4)	182 (2.8)	241.3 (618.1)
Nevada	1.4 (1.4)	3 (0.5)	2,313 (0.3)	69 (1.1)	33.5 (184.7)
New York	1.0 (1.4)	1 (0.2)	88 (0.0)	73 (1.1)	1.2 (0.7)
Ohio	2.1 (1.6)	33 (5.1)	29,862 (3.7)	356 (5.6)	83.9 (264.7)
Oregon	2.2 (1.9)	122 (18.7)	141,269 (17.3)	377 (5.9)	374.7 (727.9)
Rhode Island	1.8 (1.6)	10 (1.5)	18,130 (2.2)	57 (0.9)	318.1 (844.3)
South Carolina	1.0 (1.0)	2 (0.3)	101 (0.0)	48 (0.8)	2.1 (2.6)
Texas	0.9 (1.1)	10 (1.5)	7,552 (0.9)	418 (6.5)	18.1 (37.4)
Utah	1.7 (1.2)	3 (0.5)	663 (0.1)	66 (1.0)	10.0 (16.9)
Washington	1.9 (1.8)	20 (3.1)	18,675 (2.3)	301 (4.7)	62.0 (282.7)
Wisconsin	2.0 (1.7)	18 (2.8)	14,004 (1.7)	311 (4.9)	45.0 (159.8)

**Table:** 

State	SDI quartile no.	ZCTAs, N (%)	Multimorbidity category, N (%)		
			None, 0–1	Low, 2–<5	High, ≥5
California	1	219 (17.1)	159 (72.6)	60 (27.4)	0
California	2	346 (27.0)	231 (66.8)	111 (32.1)	4 (1.2)
California	3	310 (24.2)	175 (56.5)	134 (43.2)	1 (0.3)
California	4	407 (31.7)	243 (59.7)	162 (39.8)	2 (0.5)
Florida	1	154 (21.2)	136 (88.3)	17 (11.0)	1 (0.6)
Florida	2	221 (30.4)	194 (87.8)	27 (12.2)	0
Florida	3	194 (26.7)	164 (84.5)	29 (14.9)	1 (0.5)
Florida	4	157 (21.6)	143 (91.1)	14 (8.9)	0
Ohio	1	107 (30.1)	57 (53.3)	49 (45.8)	1 (0.9)
Ohio	2	92 (25.8)	54 (58.7)	36 (39.1)	2 (2.2)
Ohio	3	74 (20.8)	45 (60.8)	27 (36.5)	2 (2.7)
Ohio	4	83 (23.3)	42 (50.6)	40 (48.2)	1 (1.2)
Oregon	1	76 (20.1)	44 (57.9)	30 (39.5)	2 (2.6)
Oregon	2	144 (38.1)	56 (38.9)	87 (60.4)	1 (0.7)
Oregon	3	132 (34.9)	44 (33.3)	87 (65.9)	1 (0.8)
Oregon	4	26 (6.9)	7 (26.9)	19 (73.1)	0

The variance inflation factor from mixed-effects Poisson models for each patient-level covariate was less than 3, indicating no multicollinearity (Table 3). In Model 1 (aggregate SDI), residing in areas with SDI quartiles 2, 3, and 4 was associated with, respectively, a factor of 1.171 (99% CI, 1.152–1.190), 1.177 (99% CI, 1.158–1.196), and 1.180 (99% CI, 1.161–1.198) higher count of chronic diseases at baseline compared with quartile 1. We observed an increase in the count of chronic diseases per year among quartile 1 by a factor of 1.123 (99% CI, 1.121-1.126). There was a significant interaction between SDI quartile and time, indicating a lower rate of chronic disease accumulation per year (incident rate ration [IRR], 0.987; 99% CI, 0.985–0.989) for quartile 2, a decrease by a factor of 0.989 (99% CI, 0.987–0.991) for quartile 3, and a decrease by a factor of 0.998 (99% CI, 0.996–1.000) for quartile 4, all relative to the rate of increase in quartile 1.

**Table 3:** Longitudinal Mixed-Effects Poisson Regression of Chronic Disease Accumulation^a^, ADVANCE Clinical Research Network, 2012–2019

Variable	Model 1, Aggregate SDI, IRR (99% CI)	Model 2, Discrete Community-Level indicator, IRR (99% CI)
**Intercept: Baseline count of conditions**	0.381 (0.345–0.420)	0.354 (0.323–0.388)
**Time (in years)^b^: Rate of chronic conditions per patient-year**	1.123 (1.121–1.126)	1.116 (1.116–1.117)
**SDI score^c^**		
Quartile 1 (lowest deprivation area)	Reference	—d
Quartile 2	1.171 (1.152–1.190)	—d
Quartile 3	1.177 (1.158–1.196)	—d
Quartile 4 (highest deprivation area)	1.180 (1.161–1.198)	—d
**Interaction between time^b^ and SDI score^c^**		
Time*SDI Score [quartile 1]	Reference	—d
Time*SDI Score [quartile 2]	0.987 (0.985–0.989)	—d
Time*SDI Score [quartile 3]	0.989 (0.987–0.991)	—d
Time*SDI Score [quartile 4]	0.998 (0.996–1.000)	—d
**Age, y**		
45–54	Reference	Reference
55–64	1.329 (1.320–1.337)	1.328 (1.320–1.337)
≥65	1.666 (1.655–1.678)	1.664 (1.653–1.676)
**Sex**		
Female	Reference	Reference
Male	1.006 (1.000–1.011)	1.008 (1.003–1.014)
**Race or ethnicity**		
Hispanic, English preferred	0.910 (0.900–0.920)	0.910 (0.900–0.920)
Hispanic, Spanish preferred	0.820 (0.814–0.827)	0.824 (0.818–0.831)
Hispanic, other language preferred	0.731 (0.686–0.780)	0.735 (0.690–0.783)
Non-Hispanic Asian	0.797 (0.785–0.810)	0.808 (0.795–0.820)
Non-Hispanic Black	0.957 (0.948–0.965)	0.935 (0.927–0.943)
Non-Hispanic White	Reference	Reference
Non-Hispanic Other	1.014 (0.989–1.040)	1.015 (0.990–1.041)
No information	0.810 (0.801–0.820)	0.821 (0.811–0.831)
**Federal poverty level, %**		
Continuously <138	Reference	Reference
Continuously ≥138	0.770 (0.763–0.778)	0.772 (0.764–0.779)
Intermittently over or under 138	1.012 (1.004–1.021)	1.015 (1.006–1.023)
Missing	0.877 (0.870–0.884)	0.863 (0.856–0.870)
**Insurance**		
Continuously uninsured	Reference	Reference
Continuously insured	1.208 (1.197–1.219)	1.214 (1.202–1.225)
Discontinuously insured	1.477 (1.463–1.491)	1.484 (1.470–1.498)
Missing	1.170 (1.138–1.202)	1.182 (1.150–1.215)
**Urbanicity/Rurality**		
Urban	Reference	Reference
Rural	1.052 (1.043–1.061)	1.012 (1.004–1.021)
**Median annual household income, $**		
0–<56,447	—d	1.139 (1.117–1.160)
56,447–<93,994	—d	1.119 (1.099–1.140)
≥93,994	—d	Reference
**Area–level percentage unemployment, %**		
0–<8.74	—d	Reference
8.74–<19.39	—d	1.133 (1.126–1.140)
≥19.39	—d	1.264 (1.239–1.289)
**Area–level college graduate, %**		
0–<25.89	—d	1.107 (1.094–1.120)
25.89–<48.07	—d	1.038 (1.027–1.050)
≥48.07	—d	Reference

Abbreviations: ADVANCE, Accelerating Data Value Across a National Community Health Center; SDI, Social Deprivation Index; IRR, incidence rate ratio; ZCTA, zip code tabulation area.

a Accumulation is defined as the increase in an individual’s count of chronic diseases over time.

b A one-unit increase in time (in years) is associated with the respective IRR increase in count of chronic diseases over time. Asterisks indicate an interaction where SDI score quartiles can have varying slopes over time.

c SDI Score is a composite of 7 area-level variables collected by the US Census Bureau, ranging from a score of 1-100, further categorized into quartiles.

d The variable and its levels were not inserted into the model.

As expected, older age was associated with a greater number of chronic diseases. Patients aged 55 to 64 and 65 or older had, respectively, more chronic diseases compared with patients aged 45 to 54 years by a factor of 1.329 (99% CI, 1.320-1.337) and 1.666 (99% CI, 1.655-1.678). After controlling for patient-level covariates and SDI, non-Hispanic Black, Hispanic preferring English, Hispanic preferring Spanish, and non-Hispanic Asian patients all had significantly fewer chronic diseases than non-Hispanic White patients, ranging from a factor of 0.957 (99% CI, 0.948–0.965) to 0.797 (99% CI, 0.785–0.810) lower than non-Hispanic White. Patients with incomes at or over 138% of the FPL had a factor of 0.770 (99% CI, 0.763–0.778) lower chronic disease counts than those with incomes less than 138% of the FPL. Patients who were continuously insured or discontinuously insured had, respectively, a factor of 1.208 (99% CI, 1.197–1.219) to 1.477 (99% CI, 1.463–1.491) higher chronic disease counts compared with continuously uninsured patients. Patients residing in rural areas had a 1.052 (99% CI, 1.043–1.061) higher chronic disease count than patients in urban areas.

In the model containing discrete community-level indicators (Model 2), patients residing in ZCTAs with a median household income less than $56,447 had an increase in the number of chronic diseases compared with patients in ZCTAs with median income less than $93,994 by a factor of 1.139 (99% CI, 1.117-1.160). Similarly, those residing in ZCTAs with the highest level of unemployment (≥19.4%) had an increase in chronic diseases relative to the lowest level of unemployment (<8.7%) by a factor of 1.264 (99% CI, 1.239–1.289). Finally, patients in ZCTAs with the lowest level (<25.89%) of college graduates had an increase in chronic diseases compared with those in ZCTAs with the highest level of college graduates (≥48.1%) by a factor of 1.107 (99% CI, 1.094–1.120).

## Discussion

We observed a higher multimorbidity burden in more socially deprived geographic areas. This result supports previous findings that community-level social determinants of health, as indexes of social deprivation or discrete area-level features, are important drivers of health outcomes. In a nationally representative sample of adults in the UK, Knies and Kumari observed increased prevalence of multimorbidity among those living in more socially deprived areas based on the Index of Multiple Deprivation (IMD) (25). Furthermore, they observed that, among the domains assessed by the Index of Multiple Deprivation, income, employment, and education deprivation were most strongly associated with increased risk of multimorbidity. Similar findings have been reported in multiple studies in the UK and other European countries, but few studies in the US (26). Chamberlain et al found that an increased Area Deprivation Index was associated with an increased risk of multimorbidity after adjustment for individual-level socioeconomic status (27). However, that study was conducted among 7 counties in a single state, limiting generalizability. To our knowledge, ours is the first study that describes multimorbidity burden by social deprivation for a large geographic area in the US.

As expected, the number of chronic diseases increased over time among all SDI quartiles. This is consistent with extensive literature documenting increasing multimorbidity with advancing age (1,2). However, we also observed that patients in higher social deprivation quartiles had slower rates of chronic disease accumulation relative to those in the least deprived quartile. This finding could plausibly be attributed to numerous potential methodologic issues, including ceiling effects slowing the rate of chronic disease accumulation among patients with a high number of conditions at baseline and survivor bias resulting from greater attrition or mortality among those with advanced multimorbidity.

Notably, we used data from CHCs, which disproportionately serve racial and ethnic minority groups, people with fewer socioeconomic resources, and people who are uninsured or underinsured. These patients are more likely to experience social needs such as transportation barriers or food and financial insecurity, rendering them particularly vulnerable to the impact of community-level deprivation, which may compound access barriers and lead to unmet health care needs and undetected conditions (11,24). CHCs in the US have a strong focus on collecting data on social needs and providing referral to assistance services. Identifying a system to connect patients’ neighborhood conditions to social needs data could assist outreach strategies to improve chronic disease detection and management (16).

Although some previous research (28) documented increased rates of multimorbidity among people of racial and ethnic minority groups, prior work that focused on patients in CHCs found higher rates of chronic disease accrual among non-Hispanic White patients relative to other racial and ethnic groups and more frequent somatic, mental, and mental–somatic multimorbidity (12,29). However, CHC populations consist largely of patients of low socioeconomic status, including non-Hispanic White patients, which limits comparability with studies in more diverse, affluent populations (29). Our study adds to this body of work by including adjustment for key area-level characteristics associated with health outcomes. Notably, after controlling for both patient- and area-level sociodemographic indicators, we observed lower counts of chronic diseases among non-Hispanic Black, English- and Spanish-preferring Hispanic, and non-Hispanic Asian patients relative to non-Hispanic White patients. These findings, when placed in the context of previous work documenting racial and ethnic disparities in multimorbidity accumulation, highlight the unique needs, burdens, and multimorbidity distribution of this safety-net population and point to the need for greater understanding of the role socioenvironmental factors play in the development of multimorbidity among low-income and racial and ethnic minority groups.

Our finding of higher rates of multimorbidity among people continuously or discontinuously insured relative to those continuously uninsured may seem somewhat counter-intuitive but is consistent with several previous studies (30,31) and may reflect a larger reservoir of undiagnosed chronic disease among people without health insurance coverage. For example, a recent study reported a significant increase in multimorbidity rates among uninsured CHC patients newly enrolled in Medicare compared with those continuously insured before Medicare eligibility (31). Because undiagnosed, and thus unmanaged, chronic conditions result in poorer health outcomes, this points to the necessity of ensuring health coverage to reduce the prevalence of unrecognized multimorbidity. However, caution is warranted, given that increased medical encounters may also add to treatment burden, overdiagnosis, and overtreatment among older adults with multiple chronic conditions.

### Limitations

Several methodologic limitations warrant discussion. The general finding that chronic condition counts increased over time in all SDI quartiles may be due in part to changes in diagnostic criteria for several of the assessed conditions (eg, hypertension) (32). We were unable to examine severity of chronic conditions. Future research is needed to validate methods for determining chronic disease severity from EHR data. Our analysis was limited to people residing in a single state (or at a single address) throughout the study period, eliminating 1.3% of our sample. Finally, although we had data from 27 states, some states included fewer sites and patients (eg, New York had 1 site and California had 134 sites). Nevertheless, to our knowledge, ours is the largest US study of the relationship between area-level social deprivation and multimorbidity development and progression.

### Conclusion

Several recent reports and studies have called for consideration of community-level indicators in addition to patient-level social determinants of health. Area-level indicators offer valuable context and are uniformly reported across differing clinical practices, data collection procedures, and state health care policies (20,33). Linking area-level social determinants of health data to patient EHRs provides beneficial context for understanding patient risk, prioritizing treatment and outreach, and connecting to community resources at the point of care (33). Many countries use area-level deprivation scores to allocate resources (34). Prioritizing precise (or small) geographic areas that have a greater multimorbidity burden could inform programs to reduce disease progression and mitigate poor health outcomes, thus supporting the quintuple aim to improve patient experience, improve population health, reduce costs, support clinician well-being, and advance health equity (34,35). Providing sufficient supports and adequate resources to the US health care safety net will be critical to realizing the goals of the quintuple aim.

## Appendix

**Appendix Table 1:** Bivariate Analysis of Discrete Community-Level Indicators and Number of Chronic Diseases Recorded at Patient First Visit, ADVANCE Clinical Research Network, 2012–2019

Community-level characteristics	IRR (99% CI)	*P* value
**Area–level median household annual income, $**		
Intercept	0.791 (0.717–0.872)	<.001
0–<56,447	1.218 (1.195–1.241)	<.001
56,447–<93,994	1.127 (1.106–1.149)	<.001
≥93,944	Reference	
**Area–level unemployment, %**		
Intercept	0.894 (0.813–0.983)	.002
0–<8.74	Reference	
8.74–<19.39	1.121 (1.114–1.128)	<.001
19.39–100.00	1.230 (1.207–1.255)	<.001
**Area–level college graduates, %**		
Intercept	0.889 (0.807–0.978)	.002
0% – <25.89	1.083 (1.072–1.094)	<.001
25.89–<48.07	1.019 (1.008–1.030)	<.001
48.07–100.00	Reference	

Abbreviations: ADVANCE, Accelerating Data Value Across a National Community Health Center; IRR-Incident Rate Ratios.

**Appendix Table 2:** Descriptive Summary of Multimorbidity Categories by State and SDI Quartile, ADVANCE Clinical Research Network, 2012–2019

State	SDI quartile	ZCTAs, N (%)	Multimorbidity category, N (%)		
			None, 0–1	Low, 2–<5	High, ≥5
Alaska	1	6 (20.7)	5 (83.3)	1 (16.7)	0
Alaska	2	11 (37.9)	7 (63.6)	4 (36.4)	0
Alaska	3	7 (24.1)	6 (85.7)	1 (14.3)	0
Alaska	4	5 (17.2)	5 (100.0)	0	0
California	1	219 (17.1)	159 (72.6)	60 (27.4)	0
California	2	346 (27.0)	231 (66.8)	111 (32.1)	4 (1.2)
California	3	310 (24.2)	175 (56.5)	134 (43.2)	1 (0.3)
California	4	407 (31.7)	243 (59.7)	162 (39.8)	2 (0.5)
Connecticut	1	11 (50.0)	8 (72.7)	3 (27.3)	0
Connecticut	2	6 (27.3)	5 (83.3)	1 (16.7)	0
Connecticut	3	1 (4.5)	1 (100.0)	0	0
Connecticut	4	4 (18.2)	3 (75.0)	0	1 (25.0)
Florida	1	154 (21.2)	136 (88.3)	17 (11.0)	1 (0.6)
Florida	2	221 (30.4)	194 (87.8)	27 (12.2)	0
Florida	3	194 (26.7)	164 (84.5)	29 (14.9)	1 (0.5)
Florida	4	157 (21.6)	143 (91.1)	14 (8.9)	0
Georgia	1	38 (19.5)	34 (89.5)	4 (10.5)	0
Georgia	2	50 (25.6)	38 (76.0)	11 (22.0)	1 (2.0)
Georgia	3	53 (27.2)	45 (84.9)	7 (13.2)	1 (1.9)
Georgia	4	54 (27.7)	46 (85.2)	6 (11.1)	2 (3.7)
Hawaii	1	3 (6.5)	2 (66.7)	1 (33.3)	0
Hawaii	2	21 (45.7)	14 (66.7)	7 (33.3)	0
Hawaii	3	18 (39.1)	15 (83.3)	2 (11.1)	1 (5.6)
Hawaii	4	4 (8.7)	3 (75.0)	1 (25.0)	0
Indiana	1	64 (27.2)	50 (78.1)	14 (21.9)	0
Indiana	2	80 (34.0)	59 (73.8)	20 (25.0)	1 (1.2)
Indiana	3	50 (21.3)	36 (72.0)	13 (26.0)	1 (2.0)
Indiana	4	41 (17.4)	32 (78.0)	9 (22.0)	0
Kansas	1	47 (35.9)	39 (83.0)	8 (17.0)	0
Kansas	2	49 (37.4)	39 (79.6)	10 (20.4)	0
Kansas	3	26 (19.8)	20 (76.9)	5 (19.2)	1 (3.8)
Kansas	4	9 (6.9)	6 (66.7)	3 (33.3)	0
Massachusetts	1	220 (56.1)	148 (67.3)	71 (32.3)	1 (0.5)
Massachusetts	2	72 (18.4)	51 (70.8)	21 (29.2)	0
Massachusetts	3	43 (11.0)	28 (65.1)	14 (32.6)	1 (2.3)
Massachusetts	4	57 (14.5)	29 (50.9)	28 (49.1)	0
Maryland	1	59 (36.0)	40 (67.8)	19 (32.2)	0
Maryland	2	65 (39.6)	42 (64.6)	22 (33.8)	1 (1.5)
Maryland	3	22 (13.4)	12 (54.5)	8 (36.4)	2 (9.1)
Maryland	4	18 (11.0)	13 (72.2)	5 (27.8)	0
Michigan	1	27 (21.1)	16 (59.3)	11 (40.7)	0
Michigan	2	34 (26.6)	21 (61.8)	12 (35.3)	1 (2.9)
Michigan	3	30 (23.4)	16 (53.3)	12 (40.0)	2 (6.7)
Michigan	4	37 (28.9)	16 (43.2)	21 (56.8)	0
Minnesota	1	85 (49.4)	62 (72.9)	22 (25.9)	1 (1.2)
Minnesota	2	38 (22.1)	30 (78.9)	8 (21.1)	0
Minnesota	3	30 (17.4)	26 (86.7)	4 (13.3)	0
Minnesota	4	19 (11.0)	15 (78.9)	4 (21.1)	0
Missouri	1	35 (22.9)	31 (88.6)	4 (11.4)	0
Missouri	2	48 (31.4)	31 (64.6)	16 (33.3)	1 (2.1)
Missouri	3	43 (28.1)	20 (46.5)	21 (48.8)	2 (4.7)
Missouri	4	27 (17.6)	11 (40.7)	16 (59.3)	0
Montana	1	36 (42.9)	29 (80.6)	7 (19.4)	0
Montana	2	37 (44.0)	32 (86.5)	4 (10.8)	1 (2.7)
Montana	3	10 (11.9)	6 (60.0)	4 (40.0)	0
Montana	4	1 (1.2)	0 (0.0)	1 (100.0)	0
North Carolina	1	45 (13.2)	26 (57.8)	19 (42.2)	0
North Carolina	2	72 (21.1)	35 (48.6)	36 (50.0)	1 (1.4)
North Carolina	3	132 (38.6)	84 (63.6)	47 (35.6)	1 (0.8)
North Carolina	4	93 (27.2)	48 (51.6)	44 (47.3)	1 (1.1)
New Jersey	1	14 (36.8)	10 (71.4)	4 (28.6)	0
New Jersey	2	12 (31.6)	8 (66.7)	3 (25.0)	1 (8.3)
New Jersey	3	2 (5.3)	2 (100.0)	0	0
New Jersey	4	10 (26.3)	9 (90.0)	1 (10.0)	0
New Mexico	1	42 (22.8)	38 (90.5)	4 (9.5)	0
New Mexico	2	51 (27.7)	41 (80.4)	10 (19.6)	0
New Mexico	3	56 (30.4)	45 (80.4)	10 (17.9)	1 (1.8)
New Mexico	4	35 (19.0)	30 (85.7)	5 (14.3)	0
Nevada	1	22 (31.0)	21 (95.5)	1 (4.5)	0
Nevada	2	21 (29.6)	15 (71.4)	6 (28.6)	0
Nevada	3	13 (18.3)	10 (76.9)	2 (15.4)	1 (7.7)
Nevada	4	15 (21.1)	11 (73.3)	3 (20.0)	1 (6.7)
New York	1	17 (23.3)	13 (76.5)	4 (23.5)	0
New York	2	16 (21.9)	10 (62.5)	5 (31.2)	1 (6.2)
New York	3	18 (24.7)	14 (77.8)	4 (22.2)	0
New York	4	22 (30.1)	14 (63.6)	7 (31.8)	1 (4.5)
Ohio	1	107 (30.1)	57 (53.3)	49 (45.8)	1 (0.9)
Ohio	2	92 (25.8)	54 (58.7)	36 (39.1)	2 (2.2)
Ohio	3	74 (20.8)	45 (60.8)	27 (36.5)	2 (2.7)
Ohio	4	83 (23.3)	42 (50.6)	40 (48.2)	1 (1.2)
Oregon	1	76 (20.1)	44 (57.9)	30 (39.5)	2 (2.6)
Oregon	2	144 (38.1)	56 (38.9)	87 (60.4)	1 (0.7)
Oregon	3	132 (34.9)	44 (33.3)	87 (65.9)	1 (0.8)
Oregon	4	26 (6.9)	7 (26.9)	19 (73.1)	0
Rhode Island	1	21 (36.2)	19 (90.5)	2 (9.5)	0
Rhode Island	2	17 (29.3)	14 (82.4)	3 (17.6)	0
Rhode Island	3	10 (17.2)	9 (90.0)	1 (10.0)	0
Rhode Island	4	10 (17.2)	9 (90.0)	1 (10.0)	0
South Carolina	1	6 (12.5)	5 (83.3)	1 (16.7)	0
South Carolina	2	10 (20.8)	8 (80.0)	2 (20.0)	0
South Carolina	3	14 (29.2)	13 (92.9)	1 (7.1)	0
South Carolina	4	18 (37.5)	12 (66.7)	6 (33.3)	0
Texas	1	61 (14.6)	50 (82.0)	11 (18.0)	0
Texas	2	94 (22.5)	80 (85.1)	14 (14.9)	0
Texas	3	109 (26.1)	85 (78.0)	24 (22.0)	0
Texas	4	154 (36.8)	140 (90.9)	14 (9.1)	0
Utah	1	27 (40.9)	18 (66.7)	9 (33.3)	0
Utah	2	18 (27.3)	12 (66.7)	6 (33.3)	0
Utah	3	13 (19.7)	11 (84.6)	2 (15.4)	0
Utah	4	8 (12.1)	6 (75.0)	2 (25.0)	0
Washington	1	81 (26.8)	68 (84.0)	13 (16.0)	0
Washington	2	104 (34.4)	77 (74.0)	26 (25.0)	1 (1.0)
Washington	3	75 (24.8)	54 (72.0)	18 (24.0)	3 (4.0)
Washington	4	42 (13.9)	33 (78.6)	8 (19.0)	1 (2.4)
Wisconsin	1	112 (35.9)	61 (54.5)	49 (43.8)	2 (1.8)
Wisconsin	2	128 (41.0)	77 (60.2)	49 (38.3)	2 (1.6)
Wisconsin	3	47 (15.1)	27 (57.4)	20 (42.6)	0
Wisconsin	4	25 (8.0)	4 (16.0)	21 (84.0)	0

Abbreviations: ADVANCE, Accelerating Data Value Across a National Community Health Center; SDI, Social Deprivation Index; ZCTA, zip code tabulation area.
